# Supplementary material for: Platelet clearance via shear-induced unfolding of a membrane mechanoreceptor
Source: Nat Commun. 2016 Sep 27;7:12863. doi: 10.1038/ncomms12863 (PMC5052631; doi:10.1038/ncomms12863)
Supplement: Supplementary Information — Supplementary Figures 1-10. [file ncomms12863-s1.pdf]

Supplementary Figure 1:

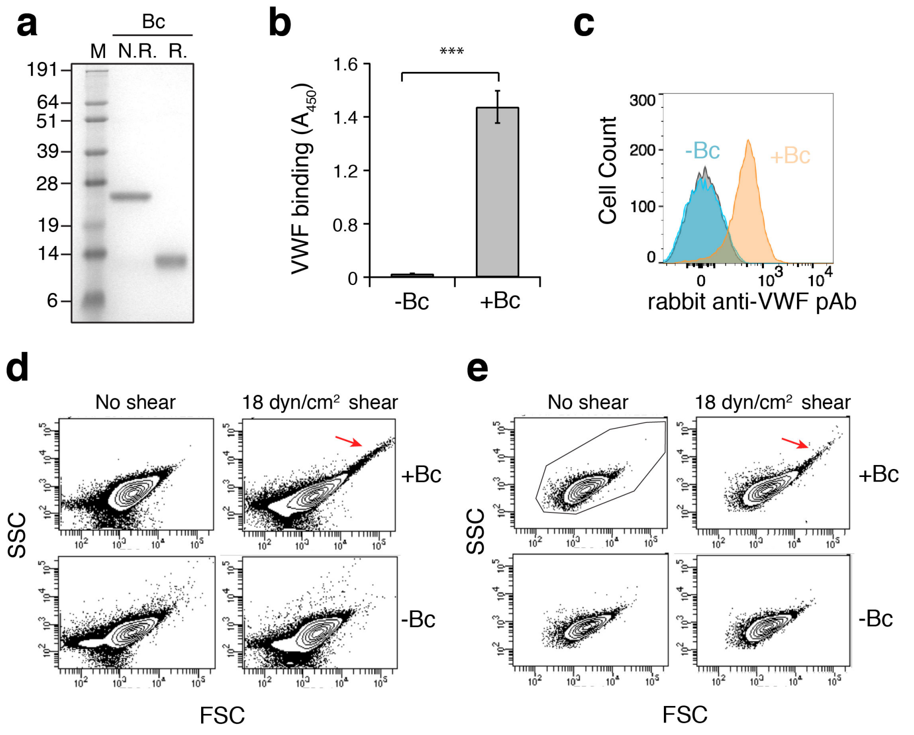

Supplementary Figure 1. Botrocetin induces binding of human VWF to human platelets in the absence of elevated shear and induces platelet agglutination as detected by flow cytometry. (a) Purified botrocetin (Bc) as stained by Coomassie blue in a 15% Bis–Tris SDS gel under nonreducing (N.R.) and reducing (R.) conditions. Molecular weight markers (M) are shown and labeled in kDa on the left. (b) Binding of VWF to immobilized GPIb-IX in the absence and presence of 2 $\mu g \cdot mL^{-1}$ botrocetin. VWF binding was detected using rabbit anti-VWF antibody and HRP-labeled donkey anti-rabbit secondary antibody. Data are shown as mean±s.d. (n=3). The *p* value is for paired Student's t-test; ***, $p<0.001$. (c) Representative flow histograms showing the binding of plasma VWF in PRP to the platelet induced by botrocetin. Human PRP was incubated with 1 $\mu g \cdot mL^{-1}$ botrocetin for 10 min at 20˚C, washed, and fixed with 4% paraformaldehyde before flow analysis. Binding of VWF was detected using rabbit anti-

VWF antibody and a fluorophore-conjugated donkey anti-rabbit secondary antibody. In the negative control, the PRP sample was incubated with only the secondary antibody. (d,e) Side scattering (SSC)/forward scattering (FSC) bivariate contour plots of human platelets (200k per μL) (d) without gating and (20k per μL) (e) with the same gating as illustrated after treatment of botrocetin and/or shear in a cone-plate viscometer as described. Red arrowheads denote the agglutinated platelets in the plots, at least those within the size limit detectable by flow cytometry.

Supplementary Figure 2:

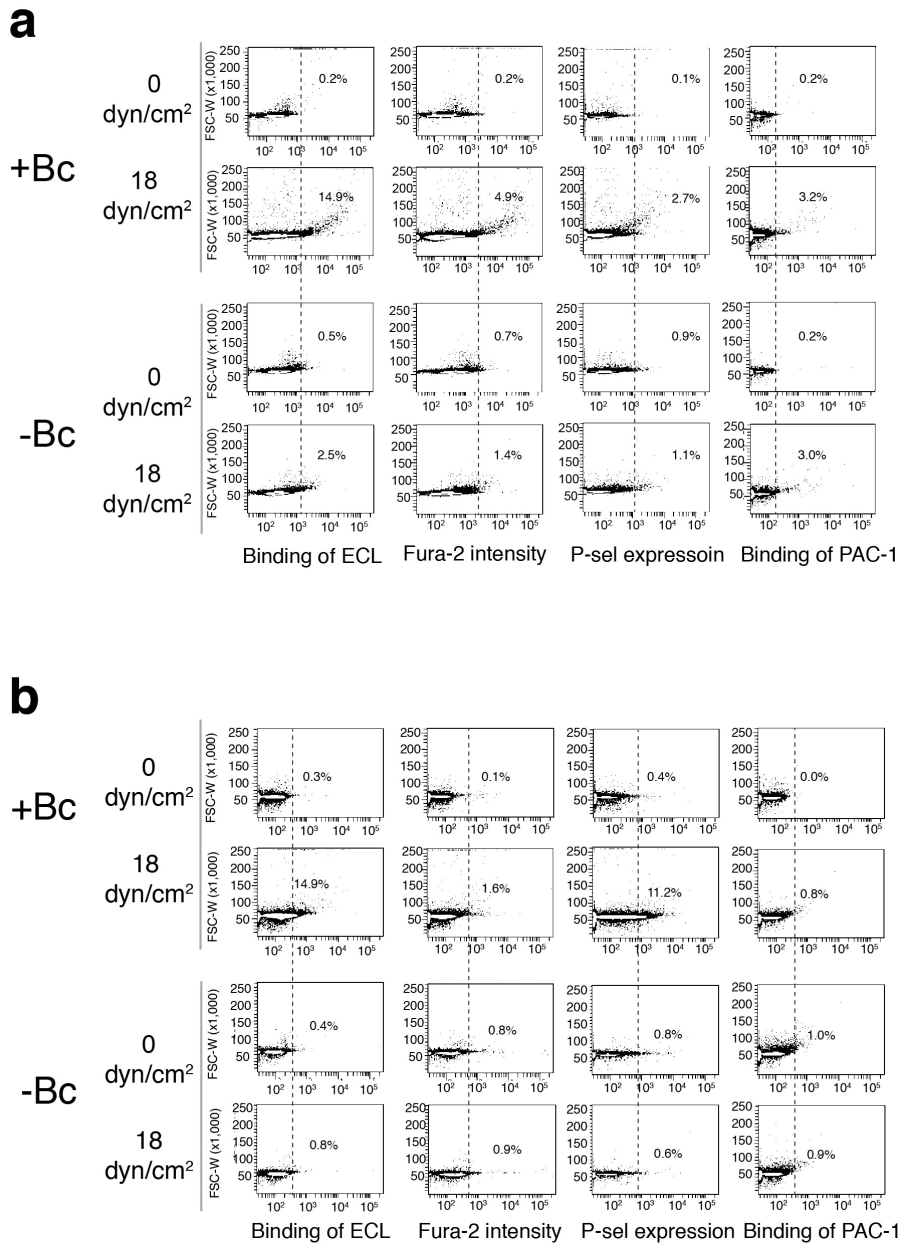

Supplementary Figure 2. Representative bivariate FSC-W/signal plots of botrocetin/shear-dependent signaling in platelets. Human PRP at platelet counts of (a) 200k per µL and (b) 20k per µL was treated at noted conditions (± 1 µg·mL^{-1} botrocetin for 10 min, then 0 or 18 dyn·cm^{-2} shear stress for 1 min) at 20 ˚C. Platelets were collected, fixed, and probed for binding of FITC-labeled ECL, Fura-2 fluorescence,

binding of anti-P-selectin antibody and binding of PAC-1 antibody by flow cytometry. Identical gating (dashed line) was applied to all the samples in the same detection group.

Supplementary Figure 3:

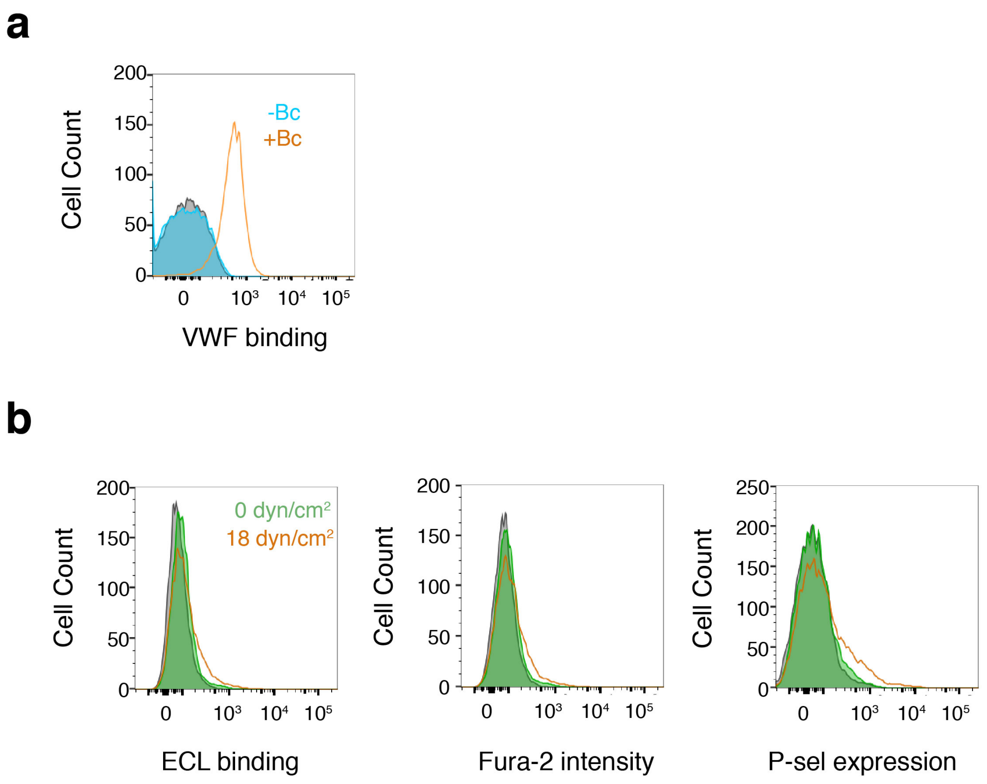

Supplementary Figure 3. Similar botrocetin/shear-induced signaling in recalcified platelets that had been pretreated with RGDS peptide. Citrated human PRP (20k per μL platelet count) was incubated with 200 μM RGDS peptide for 0.5 h at 20°C, and then recalcified to the final calcium concentration of 1 mM. Afterwards, the sample underwent the same botrocetin/shear treatment and analyzed by flow cytometry as described. (a) Representative overlaid flow histograms showing botrocetin-induced binding of plasma VWF to the platelets: turquoise (without botrocetin), open orange (with botrocetin) and gray (negative control, as described in Figure S1). (b) Representative flow histograms of botrocetin/shear-treated platelets showing the exposure of β-galactose (measured by FITC-labeled ECL), intracellular calcium influx (monitored by Fura-2 fluorescence), and expression of P-selectin (binding of anti-P-selectin antibody). The experiment was

repeated 3 times, and the quantitated signals are similar to those without calcium with similar statistical significance.

Supplementary Figure 4:

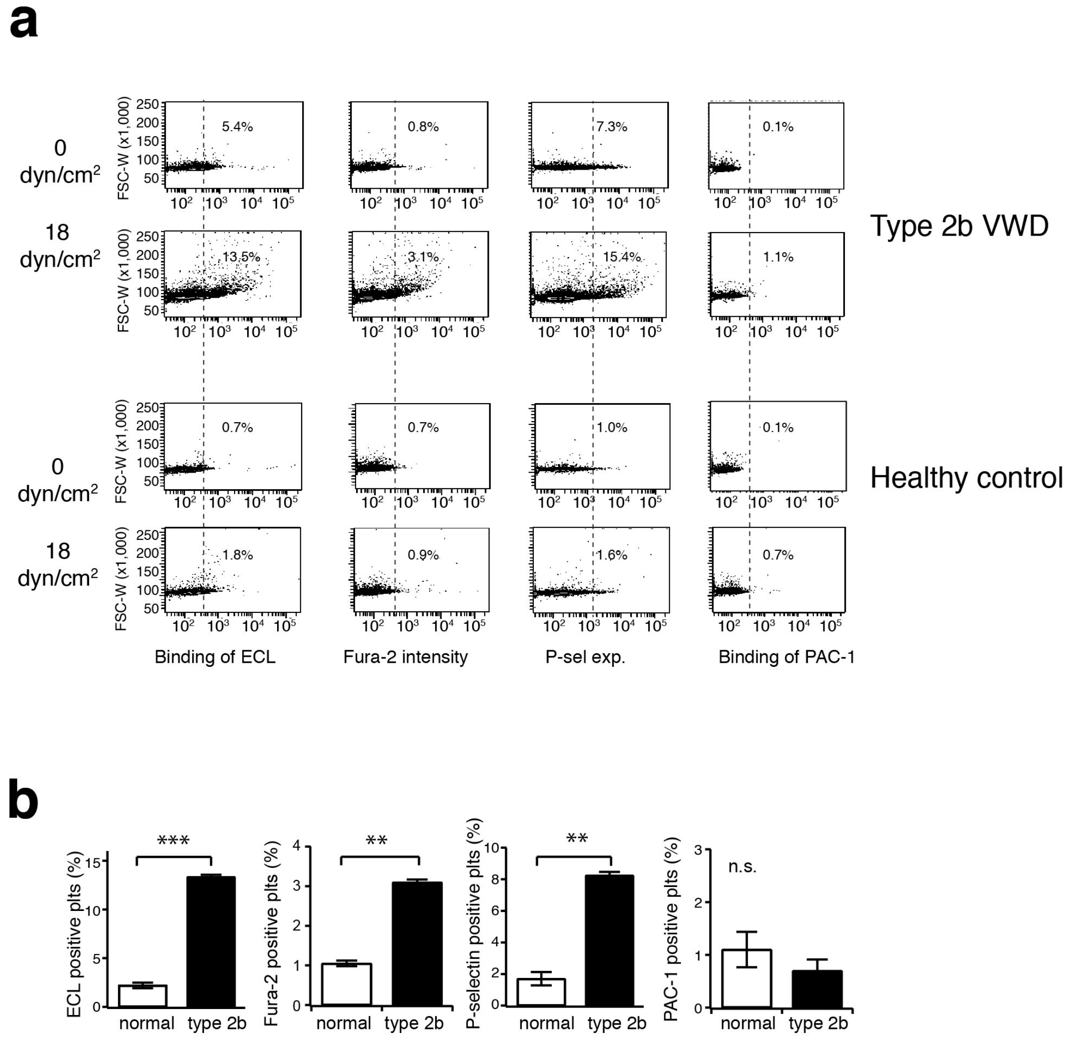

Supplementary Figure 4. Mixing type 2B VWD plasma with healthy donor platelets produces similar shear-dependent platelet signaling. (a) Plasma sample from a type 2B VWD patient (top) or a healthy donor (bottom) was mixed with healthy donor platelets at 9:1 ratio (v/v). The mixture underwent uniform shear treatment at the noted shear level, and platelet signals were detected as in Botrocetin/shear experiments. Identical gate was applied to each plot (the gray rectangle), and the percentage of cells in the gate is noted. (b) The percentage of gated positive cell population for each signaling

read-out. Data are shown as mean±s.d. (n=2). The *p* values are obtained from paired Student's *t*-test. **, $p < 0.01$; ***, $p < 0.001$; n.s., no statistical difference.

Supplementary Figure 5:

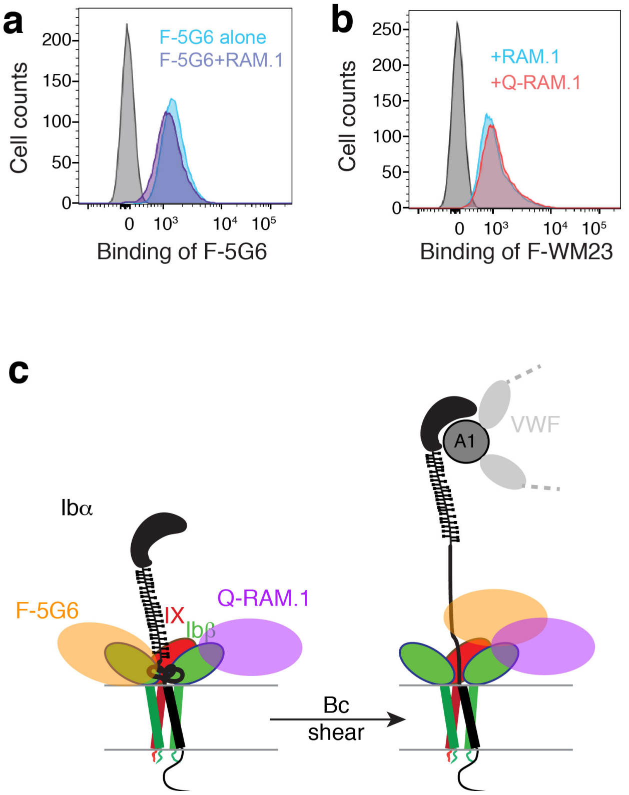

Supplementary Figure 5. Control experiments and illustration for botrocetin/shear-induced spatial change between MSD and juxtaposed GPIbβ in GPIb-IX. (a) Representative overlaid flow histograms showing the binding of fluorescently labeled 5G6 (F-5G6) is not affected by RAM.1 binding to GPIbβ. (b) Representative overlaid flow histograms showing the fluorescence of bound FITC-conjugated WM23 (F-WM23) is not quenched by quencher-labeled RAM.1 (Q-RAM.1). (c) A cartoon of the membrane-proximal parts of GPIb-IX to illustrate a likely movement of MSD induced by botrocetin/shear treatment. This movement is consistent with the botrocetin/shear-induced change in FRET efficiency between the bound 5G6 and RAM.1.

Supplementary Figure 6:

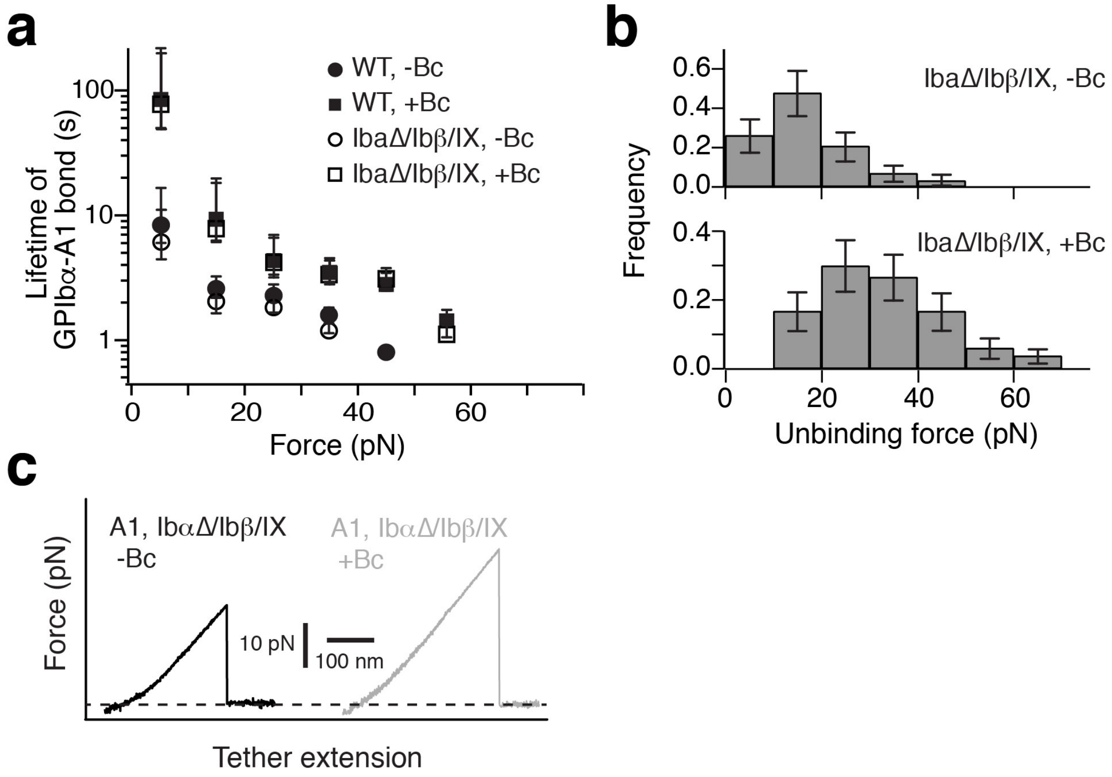

Supplementary Figure 6. The MSD-unfolding mutation does not alter binding of GPIb-IX to A1 of VWF. The binding of A1 with mutant GPIb-IX (IbαΔ/Ibβ/IX) was characterized using the same set-up as described in **Figure 2**. (a) Plot of lifetimes of the GPIb-IX/A1 bond as a function of force. Data for the wild-type (WT) complex were overlaid for comparison. Errors bars are Poisson noise. (b) Histograms of the unbinding forces between mutant GPIb-IX and A1 under a pulling rate of ~100 nm per sec. In each histogram, the Y-axis has been normalized by the total number of unbinding event (n=33-65). Errors are Poisson noise. (C) Representative force-distance traces of pulling A1 on the mutant GPIb-IX. In both presence and absence of Botrocetin, no unfolding events were observed in any of the traces.

Supplementary Figure 7:

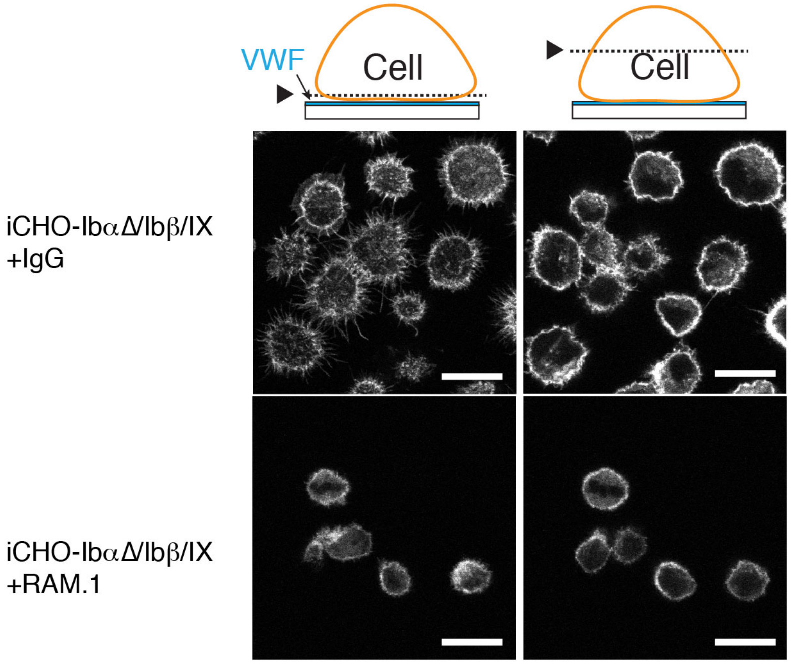

Supplementary Figure 7. Representative confocal microscopic images of VWF-attached iCHO-IbαΔ/Ibβ/IX cells pretreated with RAM.1 or rat IgG. The cells were split from the same culture, incubated with 100 nM RAM.1 or rat IgG before being applied to the VWF-coated slide and stained with FITC-phalloidin. Left: z position at the bottom of the cell; Right: z position at the middle of the cell.

Supplementary Figure 8:

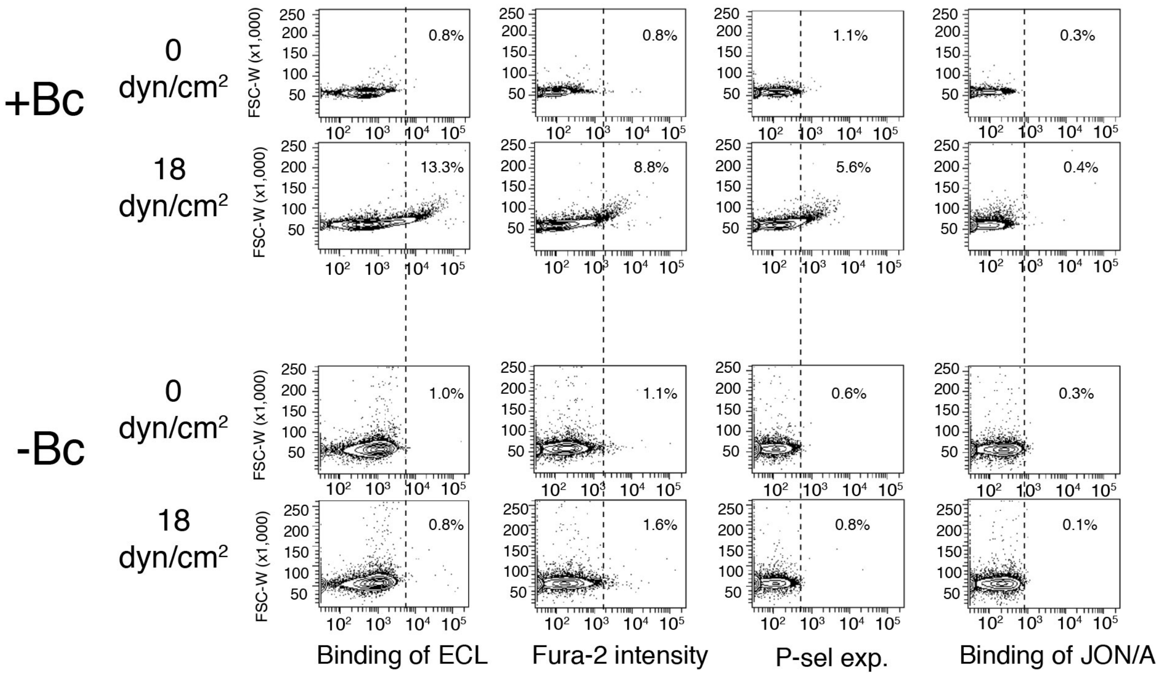

Supplementary Figure 8. Representative bivariate FSC-W/signal plots of Botrocetin/shear-dependent signaling in murine platelets. Murine PRP was treated with or without 1 $\mu g \cdot mL^{-1}$ Botrocetin for 10 min, and 0 or 18 $dyn \cdot cm^{-2}$ shear stress for 1 min at 20 ˚C. The platelet signaling was probed by binding of FITC-labeled ECL, Fura-2 fluorescence, binding of anti-P-selectin antibody and JON/A antibody. Identical gating (dashed line) was applied to all the samples in the same detection group.

Supplementary Figure 9:

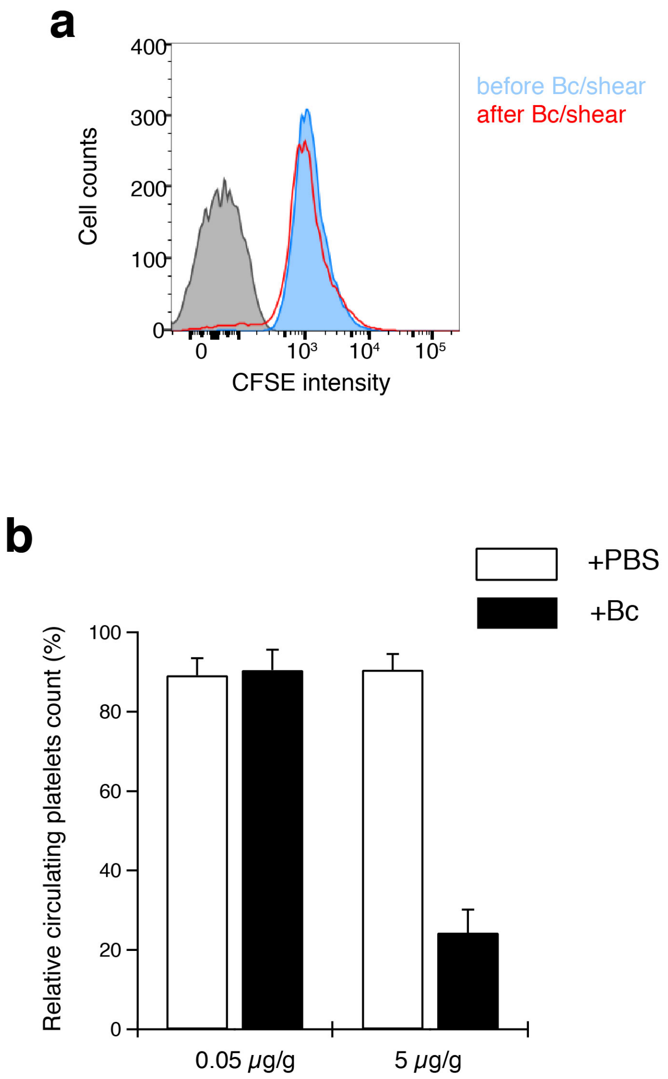

Supplementary Figure 9. Control experiments for the botrocetin-induced platelet clearance in mice. (a) Representative overlaid flow histograms showing the CFSE fluorescent intensity in the labeled murine platelets before (blue) and after (red) botrocetin/shear treatment. Gray: platelets treated with the solvent. (b) Low-dose botrocetin has no effect on mouse platelet clearance. Platelet counts in 24 hours after infusion does not reduce significantly for mice treated with 0.05 μg botrocetin per g of body weight.

Supplementary Figure 10:

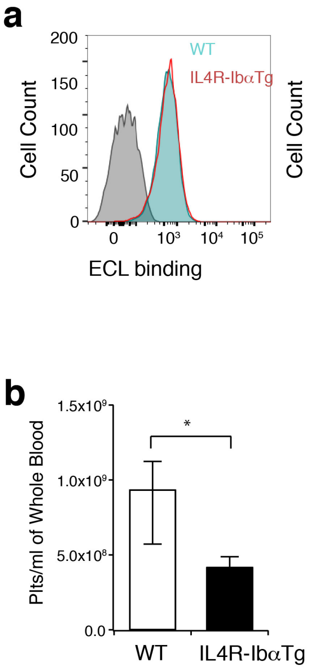

Supplementary Figure 10. IL4R-IbαTg platelets exhibit constitute GPIb-IX signaling. (a) Representative overlaid flow histograms comparing the levels of ECL binding, Fura-2 intensity and P-selectin expression between WT and IL4R-IbαTg platelets. Gray: platelets mixed with fluorophore-labeled IgG. (b) Platelet count of the IL4R-IbαTg mice is significantly lower than that of wild-type mice. Basal platelet counts are averaged from 9 IL4R-IbαTg mice and 5 wild-type mice. Data are shown as mean±SD. The *p* value is obtained from paired Student's *t*-test. *, $p < 0.05$.
